# Parent–child communication about sexual and reproductive health: evidence from the Brong Ahafo region, Ghana

**DOI:** 10.1186/s12978-015-0003-1

**Published:** 2015-03-07

**Authors:** Abubakar A Manu, Chuks Jonathan Mba, Gloria Quansah Asare, Kwasi Odoi-Agyarko, Rexford Kofi Oduro Asante

**Affiliations:** 1 Department of Population, Family, & Reproductive Health, School of Public Health, College of Health Sciences, University of Ghana, P.O. Box LG 13, Accra, Ghana; 2 Association of African Universities, P.O. Box AN 5744, Accra, Ghana; 3 Family Health Division, Ghana Health Service, Private Mail Bag, Ministries, Accra, Ghana; 4 RHI Medical Centre, Amanokrom, P.O. Box 134, Mampong-Akuapem, Eastern Region Ghana; 5 School of Applied Sciences, Central University College, Miotso Campus, P.O. Box 2305, Tema, Ghana

**Keywords:** Parent–child sexual communication, Young people, Sexual topics, Reproductive health, Ghana

## Abstract

**Background:**

Young people aged 10–24 years represent one-third of the Ghanaian population. Many are sexually active and are at considerable risk of negative health outcomes due to inadequate sexual and reproductive health knowledge. Although growing international evidence suggests that parent–child sexual communication has positive influence on young people’s sexual behaviours, this subject has been poorly studied among Ghanaian families. This study explored the extent and patterns of parent–child sexual communication, and the topics commonly discussed by parents.

**Methods:**

A cross-sectional design was used to sample 790 parent–child dyads through a two-stage cluster sampling technique with probability proportional to size. Interviewer-administered questionnaire method was used to gather quantitative data on parent–child communication about sex. Twenty sexual topics were investigated to describe the patterns and frequency of communication. The Pearson’s chi-square and z-test for two-sample proportions were used to assess sexual communication differences between parents and young people. Qualitative data were used to flesh-out relevant issues which standard questionnaire could not cover satisfactorily.

**Results:**

About 82.3% of parents had at some point in time discussed sexual and reproductive health issues with their children; nonetheless, the discussions centered on a few topics. Whereas child-report indicated that 78.8% of mothers had discussed sexual communication with their children, 53.5% of fathers had done so. Parental discussions on the 20 sexual topics ranged from 5.2%-73.6%. Conversely, young people’s report indicates that mother-discussed topics ranged between 1.9%-69.5%, while father-discussed topics ranged from 0.4% to 46.0%. Sexual abstinence was the most frequently discussed topic (73.6%), followed by menstruation 63.3% and HIV/AIDS 61.5%; while condom (5.2%) and other contraceptive use (9.3%) were hardly discussed. The most common trigger of communication cited by parent–child dyads was parent’s own initiation (59.1% vs. 62.6% *p* = 0.22).

**Conclusions:**

Parents in the Brong Ahafo region of Ghana do talk to children about sex, but their conversations cover limited topics. While abstinence is the most widely discussed sexual topic, condoms and contraception were rarely discussed. Sex educational programmes ought to encourage parents to expand sexual communication to cover more topics.

## Background

Young people aged 10–24 years face multiple challenges during their transition to adulthood. Even though the transition from childhood to adulthood lasts about 15 years, many young people could acquire significant preventable health problems before reaching adulthood. Most of those problems could persist throughout their adult life. One of the reasons for this problem is lack of adequate and accurate knowledge about sexual matters. Consequently, risky sexual behaviours such as unprotected sex, multiple sexual partnerships, and transactional sex are common among young people [1,2]. These behaviours predispose young people to the triple tragedy of sexually transmitted infections, including HIV/AIDS, unwanted teenage pregnancy and unsafe induced abortion [3,4].

Parents play a critical role in the growth, development and sexual socialization of their children. Parental involvement through parent–child sexual communication (PCSC) presents education about sex and reproductive health to young people. Studies from the developed world [5-8], and sub-Saharan Africa [1,9,10] have been unequivocal regarding parents being the most dominant sex educators. In parent–child sexual communication, parents transmit sexual values, beliefs, information and expectations to their children with the aim of influencing sexual behaviours, attitudes and decision-making of their children [11-13]. Therefore, parental sexual communication to empower young people to manage the many challenges associated with youthfulness cannot be underestimated.

There is evidence that young people prefer to receive sexual information from their parents [14], yet only a few obtain such information from them [1,15]. Research suggests that sexuality communication can be a very useful intervention that encourages sexual responsibility among young people when the message is properly and comprehensively delivered [16].

In Ghana, parent–child sexual communication has not been well explored. The search of the literature revealed that very few studies had been conducted on parent–child sexual communication, including only four (4) published ones [17]. These studies were all selective in scope, coverage and assessment. For example, Adu-Mireku [18] only examined family communication about HIV/AIDS while Kumi-Kyeremeh and colleagues [19] investigated sexual communication by characterizing persons who have talked about sex-related matters with the adolescent.

In addition, most of these Ghanaian studies sought information from the perspective of young people only; the views of their parents were not considered. However, the views of both parents and children are critical for a balanced and comprehensive assessment of sexual communication.

Presently, very little is known in Ghana about studies that targeted both parents and their children to holistically examine sexual communication between parents and young people. Specifically, there is dearth of data on the type of topic discussed and frequency of parent–child sexual communication. PCSC was therefore explored to investigate the following question:

- To what extent do parents in the Brong Ahafo region communicate with their children about sexual and reproductive health issues?
- What are the possible topics parents are most likely to discuss in their sexual communication engagement with the children?
- What are the triggers of parental sexual communication?
- Is there any difference between a global single-item (Yes/No) measure of occurrence of communication and topic-by-topic assessment?

We hypothesized that the proportion of parents who communicate with young people would not be greater than 50%; and that parents are more likely to report higher proportion of communication compared to young people’s report.

## Methods

### Study settings and period

Data for this study came from a cross-sectional study conducted between January to March 2010, involving parents and their children aged 10–24 years in the Brong Ahafo region of Ghana. The region, located at the centre of the country, and lies within longitude 0°15″E to 3° W and latitude 8°45″N to 7°30″S. It has a population of 2,310,983, of which 55.5% is rural [20]. The region shares boundaries with five other regions: the Northern region to the north, the Ashanti and Western regions to the south, the Volta Region to the east, and the Eastern region to the southeast. Also to the western frontier is La Cote d’Ivoire. Young people are about 761,179 (32.9% of the total population of the region).

### Participants

The study population was young people aged 10–24 years and their biological parents or parent-figures^a^*de facto* residents in private homes in the region.

### Inclusion/exclusion criteria

Study subjects were eligible to participate in the study if parent and child resided within a selected enumeration area; both parent and child pairs providing voluntary consent; aged between 10 years and 24 years; being either the biological parent or a parent figure who must have stayed continuously with the child for at least 2 years prior to the survey. On the contrary, participants who were on a visit to the enumeration area during the data collection period. In addition, young people who were married were considered ineligible in the study.

#### Sample size calculation

The sample size was determined with the following parameters. Prevalence of parent–child sexual communication (p) was unknown, due to lack of appropriate information on sexual discussion from previous related studies in Ghana. Thus we assumed communication prevalence in the region will be about 50%. We further assumed a 95% confidence level (z), a margin of error (d) of 5% and a design effect (Deff) of 2, in order to improve the loss of precision arising from cluster sampling technique. Thus a minimum sample size of 768 was calculated using the population proportion formula follows:

$$ N=\frac{{\left({z}_{1-\mathrm{x}/2}\right)}^2p\left(1-p\right)}{d^2}\times Deff $$

However, field data collection yielded 840 parent–child dyads, but 50 pairs of respondents were excluded for various reasons (refusal, incomplete information and being married), leaving 790 pairs with complete data for analysis.

#### Sampling procedure

The study employed both quantitative and qualitative methods. The sampling frame was the updated census EAs that were constructed by the Ghana Statistical Service for the 2000 Population and Housing Census in Ghana. The EAs consisted of or a section(s) of a town or a city, and in rural areas, one, two, or three adjoining smaller villages. Each EA was considered as a cluster, and was stratified into rural and urban components.

### Sampling for the quantitative component

The quantitative component was a household-based survey. A two-stage stratified sampling technique was used. In the first stage, 52 EAs were selected across the 22 districts from a sampling frame consisting of 2,673 EAs in the region through a systematic procedure with probability proportional to size. The measure of size was the number of households in the EA.

The second stage entailed the selection of participants from households through the modified random walk method as had been used in previous studies [21,22] as follows: EA maps and their descriptions were used to identify the boundaries of each EA. Thereafter, the various corners or key landmarks describing the EA were noted and numbered. These numbers were written on pieces of papers and one was chosen randomly. The corresponding corner/landmark was used as the random starting point. We selected 15–17 households per EA in conformity with previous sampling strategy employed by the Ghana Demographic and Health Survey [23]. After the first house/household, the next nearest house/household with eligible subjects was chosen for interview. This process continued until the target sample size for the EA was obtained.

### Selection of parent–child pairs

Young people were the index respondents; and were used as leads for selecting parents. After determining a random starting point, the nearest even or odd-numbered house (EA specific) was entered and all young people aged 10–24 years were identified alongside their parents. In each house, only one household, that is, parent–child pair was eligible for interview. Therefore, all selected single-household houses with eligible young person automatically qualified for interviews. However, in houses with multiple households (for example compound houses), one household was randomly selected for interviews.

### Selection of mothers and fathers

Both mothers and fathers were involved in the study; but in each household, only one of them was interviewed. Generally, the sex of parent was selected consecutively from one household to the other irrespective of the sex of the child. However, some fathers were difficult to reach as had been noted by previous studies [24]. In such cases, the mother was automatically interviewed, resulting in higher mother participation.

### Data collection procedures

In each household, separate confidential interview was conducted in a convenient place for the parent–child dyad to avoid eavesdropping and to ensure openness and truthful responses. Parents were generally interviewed first, followed by the index child. This procedure enabled young people to freely discuss vital issues about themselves, and also ensured they were not apprehensive of parental presence. These measures ensured that interviewer-child interactions were devoid of possible information divulge, and also to minimise socially desirable response from children.

### Sampling for the qualitative component

In-depth interview respondents were selected randomly from 13 out of the 22 districts. They were selected from the same EAs in which quantitative sample was drawn. In each selected EA, one household was selected randomly, and 26 parent–child participants who were not part of the quantitative survey were interviewed to elicit in-depth qualitative information.

### Data and information sources

Structured questionnaires were used to obtain quantitative data from parent–child dyads. Separate questionnaires solicited seemingly similar information from parents and young people. The questions were mainly extracted from previous related studies conducted mainly in developed countries including [25-31]. Questions that were alien to the Ghanaian cultural standards were adapted with cultural appropriateness. Just a few questions were developed from literature by the researchers.

The sets of questionnaires and the interview guides were forward and back translated from English to Twi (local language) by linguistics experts from the Linguistics Department of the University of Ghana. Amendments were made as necessary until final versions were obtained.

### Measures

#### Socio-demographic information

Both parent and young people were asked directly to supply socio-demographic information such as age, sex, marital status, educational level, religious affiliation, employment status and family structure. In addition, young people were asked to provide information on how long they have stayed with their parents, closeness to parents, parent restriction and parent knowledge of child’s whereabouts.

#### Sex-related topics

The study explored parent–child communication about 20 specific sex-related topics. Both parents and young people were asked to indicate whether they had ever had discussion on any of the twenty (20) specific sexual topics. The topics were categorized under three themes in reference to the procedure used by [26], namely: (1) biological/developmental (2) Sexual risk prevention or safety, and (3) experiencing sex.

#### Communication about specific sexual topics

Parents were presented with statements that asked whether they had ever talked to their children about 20 specific sex-related topics. The stem of the statements was “have you ever talked to your child about [topic] e.g. abstinence? The same statements phrased appropriately for young people sought to find out whether their parents had discussed the 20 sexual topics with them. The stem of the questions to young people was “has your mother/father ever talked to you about [topic] e.g. HIV/AIDS. Response options for both dyads were *0 = never, 1 = once, 2 = a few times, and 3 = often.* For the purposes of this analysis the responses from both parents and young people on these 20 items were recoded as *0 = never* [Never] and 1 = ever [once, a few time, and often]. Thereafter, frequencies were computed for each the 20 topics separately for parents on one hand, and young people, comprising communication with mothers and fathers on the other hand.

### Prevalence of parent–child sexual communication

The extent of sexual communication between parents and their children was assessed using two measures: (1) global measure and (2) detailed examination of 20 specific sexual topics (overall measure of communication). These procedures were used to facilitate holistic assessment of parent–child dyads’ understanding of sexual communication. We assessed reports of parents and young people separately.

### Global measure of PCSC

A direct global single-item (Yes/No) question was used to assess parent–child sexual communication. Parents were asked whether they had ever discussed sex-related matters with their child while young people were asked whether their parents had ever discussed sex-related matters with them.

### Overall measure of PCSC

Three derived variables [(1) parent ever talk; *child report of* (2) mother ever talk and (3) father ever talk] were used to assess the overall prevalence of communication using parents’ and young people’s responses to the 20 sex-related items as described above. Parents were asked to report on how much they have talked to their children about 20 specific sex-related topics, while young people were asked to report how much their mothers and fathers had talked to them on the same topics. Response options (*0 = never, 1 = once, 2 = a few times, and 3 = often*) for both dyads were converted into *0 = never* [Never] and 1 = ever [once, a few time, and often]. Different frequencies were run for these three groups. For the purposes of this study, a greater emphasis was placed on parents’ report of sexuality communication since our prime interest was to measure parental efforts at sexual communication with their children. Children reports of communication were used to validate parental report.

### Triggers for initiating talks

A single item phrased appropriately for parents and young people was used to find out key motivating factors that drive parents to initiate sexual communication action with their children. Parent–child dyads were asked directly to report the circumstance that led to sexual communicate. Response options were “Event-driven”, “suspicion of sexual activity”, “child asked a question”, and “parent’s own initiative”.

### Ethical considerations

The study received ethical clearance from the Ghana Health Service Ethical Review Committee. Informed consent was obtained from all participants after the objectives and the methodology of the study had been explained to them. For literate participants, written informed consent was sought, while witnessed verbal consent was obtained from illiterate participants. Before obtaining consent, participants were assured of privacy and confidentiality, and voluntary participation was also stressed. For young people aged 10–17 years (minors), both parental consent and minor’s assent were obtained before they participated in the study.

### Data management and analysis

The quantitative data were independently captured by two trained data entry clerks using EpiData software version 3.1. Data entry screens with appropriate variable definitions, consistency checks and skipping patterns were designed separately to capture parent’s and young people’s questionnaires. The two entries were validated for accuracy and then exported from EpiData environment to IBM SPSS Statistics 20 (IBM Corp, Armonk, NY) and Stata 11.0 (Stata Corp, College Station, TX). We performed further cleaning and checks to ensure absolute data integrity.

Data analyses were performed with IBM SPSS Statistics 20 and Stata 11.0. We used frequency distributions and cross-tabulations to summarize socio-demographic data, the number of sexual topics discussed by parent–child dyads. The Pearson’s Chi-square (*χ*^2^) was used to examine differences in reports of sexual communication among parents and young people by sex as well as age groups. We assessed the level of agreement between parent–child dyads by performing a correspondence analysis using kappa statistics. Parent–child responses per each sexual topic were matched to determine dyads who agreed in responses as opposed to those who differed. We applied the benchmark developed by Landis and Koch, who maintain that agreement between two responses can be considered substantively significant when kappa reaches 0.4 [32]. This benchmark has been applied in previous parent–child study to undertake assessment of parent–child agreement [33]. Two comparisons were made between parent and child report of mother communication, and parent and child report of father communication.

Additionally, a series of two-sample z-test of proportions were conducted to determine whether there were significant differences between (a) the estimated prevalence of communication vs. actual prevalence of communication as reported in the present study, (b) Global measure of communication vs. topic-by-topic assessment of communication (c) parent report vs. child report of mother communication, (d) parent report vs. child report of father communication, and (e) various proportions reported separately by parents and children about the triggers of communication. In all statistical procedures, a *p*-value of 0.05 was used to determine statistical significance.

## Results

### Distribution of the study participants

A summary statistics of socio-demographic characteristics of the 790 parent–child dyads are reported in Table 1. The study comprised 53.9% rural and 46.1% urban residents. More than half (56.2%) of the parent sample were mothers, and the about 77% were married. Parents’ ages ranged from 26 years to 76 years, with a mean age of 47.1 years (*SD* = 10.0). Fathers were 4 years older than mothers (*p* < 0.001). About two-thirds of the parents attended some school, of which half (50.5%) ended at the basic level.

**Table 1:** **Socio-demographic characteristic of parents and young people**

Characteristics	Parent			Young people		
	Total (N = 790)	Mother (N = 444)	Father (N = 346)	Total N = 790)	Daughters (N = 405)	Sons (N = 385)
Age in years, *M (SD)*	47.1 (10.0)	45.2 (10.3)	49.5 (9.2)***	16.7(4.0)	16.6 (3.8)	16.8 (4.0)
**Place of residence %**						
Rural	53.9	49.8	59.2*	53.9	50.9	57.1
Urban	46.1	50.2	40.8	46.1	49.1	42.9
**Educational level %**						
No Education	32.9	39.9	24.0***	2.5	4.0	1.0*
Basic	50.5	48.0	53.8	76.1	77.0	75.1
≥Secondary	16.6	12.2	22.3	21.4	19.0	23.9
**Religious affiliation %**						
Christian	78.6	82.4	73.7**	81.0	83.0	79.0
Muslim	13.0	12.4	13.9	12.4	12.1	12.7
Traditionalist	1.3	0.5	2.3	0.5	0.5	0.5
No religion	7.1	4.7	10.1	6.1	4.4	7.8
**Family structure %**						
Both parents	67.2	53.8	84.4***	67.2	62.7	71.9***
Mother only	21.1	36.0	2.0	21.1	25.2	16.9
Father only	5.1	1.1	10.1	5.1	2.7	7.5
Grandparents	6.6	9.0	3.5	6.6	9.4	3.6
**Marital status %**						
Single	2.5	2.7	2.3***			
Married	77.5	68.7	88.7			
Divorced/separated	19.0	26.8	9.0			
Cohabiting	1.0	1.8	0.0			
**Occupation %**						
Unemployed	5.2	7.7	2.0***			
Farmer	53.3	47.1	61.3			
Trade/artisan	25.5	32.4	18.8			
Formal sector	15.1	12.8	17.9			
**Age group in years %**						
10–14				33.4	32.8	34.0
15–19				39.1	41.7	36.4
20-24				27.5	25.4	39.6
**Stayed with parents %**						
Continuous stay				85.3	81.7	89.6**
Partial stay				14.7	18.5	10.6

***Note***: Statistical test compares differences between mothers and fathers for each variable. *P < 0.05. **P < 0.01. ***P <0.001.

The young people sample comprised 52% females. Young people’s ages ranged from 10 years to 24 years with a mean age of 16.7 years (*SD* = 4.0). There was no statistically significant age different between daughters and sons. About one-third (33.4%) of young people were young adolescents aged 10–14 years, 39.1% older adolescents 15–19 years old, and 27.5% young adults aged 20–24 years. About two-thirds (67.2%) live with both parents.

### Parent–child sexual communication

The extent of sexual communication between parents and their children was assessed using a global measure of sexual communication as well as detailed examination of 20 specific sexual topics. These procedures were used to facilitate holistic assessment of parent–child dyads’ understanding of sexual communication. We assessed reports of parents and young people separately.

### Global measure of parent–child sexual communication

Using a global single-item (Yes/No) measure of parent–child sexual communication, 74.4% of parents indicated they had ever communicated with their children on sexual issues while 72.8% of young people reported that their parents had ever talked to them about sexual issues. Parental sex difference was not statistically significant, but among young people, more daughters than sons reported discussion with their parents (76.3% vs. 69.1%, *p* < 0.05.). When asked whether it was important for parents to talk to them about sexual and reproductive health issues, almost all young people (98.8%) answered in the affirmative.

### Overall measure of sexual communication

**Figure 1:**
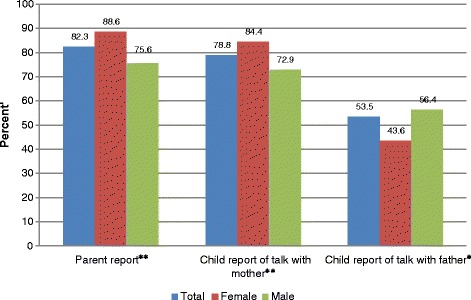
**Parents and young people sex differences in report of overall communication prevalence.***Note*: Chi-square compares differences between females and males by communication.*p < 0.01, **p < 0.001.

Assessment of the 20 specific sexual topics revealed that about 82.3% (650/790) of parents had ever talked^b^ about sexual issues with their children. Child report showed that 78.8% (617/783) of mothers had discussed sexual communication while 53.5% (374/699) of fathers had talked to their children about sex as shown in Figure 1. A two-sample z-test of proportions found significant difference between the 50% assumed prevalence of parental communication and actual (82.3%) prevalence of parent–child communication (mean difference = 0.323; 95% CI [0.279 – 0.367], *p* < 0.001). Thus the null hypothesis was rejected. Furthermore, the reports of communication from parents and by children (mother and father) were compared using z-test. A test of proportions between parents reported prevalence of communication and child report of father communication was statistically significant (mean difference = 0.288; 95% CI [0.242 – 0.0.334], *p* = 0.079). However, there was no difference in proportions between parents reported communication and child report of mother communication (mean difference = 0.035; 95% CI [−0.004 – 0.074], *p* = 0.079).

Chi-square tests showed significant differences between mothers and fathers, as well as daughters and son’s report of communication.

### Comparison between global measure of PCSC and overall topic analysis measure

We used a two sample z-test of proportions to evaluate the difference in proportions between the frequency of global communication and frequency of the overall topic analysis. We found a statistically significant difference between the frequency of communication using global measure and the overall topic analysis measure (mean difference = 0.079; 95% CI [0.038 – 0.119], *p* < 0.001. This clearly suggests that global measure of communication reports a lower amount of communication.

### Triggers of parental sexual communication

**Figure 2:**
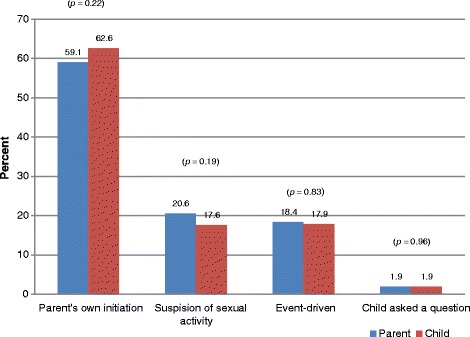
**Comparison of reason why parents initiated sexual talks as reported by parents and young people.***Note:* The figures in parenthesis on top of each pair of bars represent *p*-values for the difference in proportions for each pair of parent-child responses.

Figure 2 presents the distribution of the various triggers of parental sexual communication as reported by parents and children. Parent–child pairs were independently interviewed and responses were compiled and compared reason-by-reason. Figure 2 presents reasons that triggered parents initiate sexual talks with their children. Comparison of the two sets of responses from parents and children reveals a general trend. Independently, more than half of parents (59.1%) and children (62.6%) indicated that sexual communication was triggered on parent’s own initiative [Figure 2]. A *z-*test of proportions revealed no differences in reports from parents and children. The *p-*values for the various response categories are reported on-top of each pair of bars [Figure 2].

Table 2 presents Chi-square analyses examining parent and child reports of triggers of sexual communication selected background variables of child. The results show that child’s age and connectedness to parents were significantly associated with both parent and child reports of triggers of communication. Both reports indicate that parents are more likely initiate sexual talks on their *own initiative* with young adolescents 10–14 years compared with young people aged 20–24 years. In addition, sex of child and length of stay with parents were related with triggers of communication from child viewpoint (Table 3).

**Table 2:** **Association between parent and child perspectives on the triggers of sexual communication by selected background characteristics of child**

Variable	Parent’s own initiation		Suspicion		Event driven		Child asked a question	
	Parent	Child	Parent	Child	Parent	Child	Parent	Child
**Sex of child**	*	*		*		*		*
Female	58.6	57.6	21.3	19.4	18.8	20.1	1.9	2.9
Male	59.7	68.4	19.8	15.4	18.4	15.4	1.9	0.8
**Age group**	***	***	***	***	***	***	***	***
10-14	72.3	84.9	5.0	4.1	19.9	9.6	2.8	1.4
15-19	52.3	56.6	24.8	20.1	20.5	21.7	2.3	1.6
20-24	58.5	52.8	26.6	25.0	14.4	19.4	0.5	2.8
**Parent connectedness**	*	*	*	*	*	*	*	*
Not close	40.0	37.5	20.0	25.0	40.0	37.5	0.0	0.0
Somewhat close	54.9	58.4	23.2	21.5	21.5	20.1	0.4	0.0
Very close	62.5	65.8	18.9	14.9	15.7	16.1	2.9	3.2
**Place of residence**								
Rural	58.7	61.9	22.2	19.5	18.1	17.9	1.0	0.7
Urban	59.6	63.4	18.8	15.3	18.8	17.9	2.9	3.4
**Stay with parent**		**		**		**		**
Partial stay	59.0	54.7	22.9	23.3	15.7	16.3	2.4	5.8
Continuous stay	59.1	64.0	20.2	16.6	18.8	18.2	1.8	1.2

***Note***: Statistical test compares differences between mothers and fathers for each variable. *P < 0.05. **P < 0.01. ***P <0.001.

**Table 3:** **Parent and young people reports of discussion of specific sexual topics and the level of agreement in both reporters**

Sexual topic	Parent report	Young people report			
	Discussion with child N = 781-790*	Mother discussion N = 780-783*		Father discussion N = 690-696*	
	n (%)	n (%)	Kappa^‡^	n (%)	Kappa^‡^
***Biological/developmental***					
**Physical development**	**436 (55.5)**	**396 (50.6)**	**0.53**	**126 (18.2)**	**0.24**
**Menstruation/wet dream**	**260 (63.3)**	**298 (38.4)**	**0.51**	**43 (6.2)**	**0.02**
**Puberty**	**443 (56.3)**	**413 (52.8)**	**0.53**	**123 (17.7)**	**0.23**
Reproduction/having babies	51 (6.5)	48 (5.8)	0.42	3 (0.4)	−0.01
Masturbation	18 (2.5)	15 (1.9)	0.04	7 (1.0)	0.25
***Sexual risk prevention or safety***					
**Prevention of STDs**	**445 (56.3)**	**380 (48.7)**	**0.53**	**194 (27.9)**	**0.33**
**Prevention of HIV/AIDS**	**485 (61.5)**	**421 (53.8)**	**0.54**	**228 (32.8)**	**0.32**
**Abstaining from sex until marriage**	**581 (73.6)**	**544 (69.5)**	**0.60**	**320 (46.0)**	**0.34**
Use of condoms	73 (9.3)	68 (8.7)	0.45	35 (5.1)	0.29
Contraceptives	41 (5.2)	39 (5.0)	0.45	7 (1.0)	0.22
Pregnancy	66 (8.4)	82 (10.5)	0.36	14 (2.0)	0.09
Abortion	168 (21.3)	167 (21.3)	0.62	72 (10.2)	0.41
**Consequences of premarital sex**	**380 (48.3)**	**357 (45.6)**	**0.69**	**213 (30.6)**	**0.48**
**Substance use**	**315 (39.9)**	**302 (38.6)**	**0.63**	**199 (28.6)**	**0.56**
***Experiencing sex***					
Sexual feelings	51 (6.5)	35 (4.5)	0.26	9 (1.3)	0.10
When to start sexual intercourse	150 (19.0)	127 (16.2)	0.60	61 (8.8)	0.37
Choosing sexual partners	90 (11.4)	77 (9.8)	0.56	27 (3.7)	0.43
How to handle sexual pressure	59 (7.2)	48 (6.1)	0.41	9 (1.3)	0.22
Safer sex	62 (7.9)	54 (6.9)	0.52	14 (2.0)	0.26
Homosexuality	42 (5.2)	37 (4.7)	0.56	29 (4.2)	0.47

***Note*:** *Sample sizes for the various topics vary due to sporadic missing values.

^‡^Kappa compares agreement between (a) parent report vs. child’s report of mother communication, and (b) parent report vs. child’s report of father communication.

In-depth interview with parents and young people confirmed that more parents engage children in sexual talks largely on parents’ own initiation. The following statements support the overwhelming reason that parental sexual communication is started on parent’s own initiative:

*“…because she is growing into womanhood, she must know and be able to imitate me as a mother, so on my own accord had to teach her things relating to her sexual life as well as household chores” (Urban mother, aged 47 years).*

*“Premarital sex brings hardships in the family, and girls suffer most compared to boys…, so it is very important to let them know about these things (sexual issues), because if you do not tell them they will go out there and bring you all sorts of irresponsible behaviours with their untold hardships” (Rural mother, aged 38 years).*

*“My daughter looks older in stature, so I felt she should know about these things…so that no man can deceive her” (Rural mother, aged 36 years).*

*“In the olden days, young people were able to abstain from sex until marriage, but these days, no! So I advised him to emulate me and stay away from girls and bad boys” (Rural father, aged 60 years).*

### Commonly discussed sexual topics by parents

Presented in Table 3 are the proportions of parent–child dyads reports of discussion on each of the 20 specific sexual topics. Parental discussions with young people on specific topics ranged from 5.2% - 73.6%. On the other hand, young people’s report indicates that mother-discussed topics ranged between 1.9% and 69.5%, while father discussed topics ranged from 0.4% to 46.0%. Based on the proportions, four main patterns of PCSC are clearly discerned, First, parents’ tended to report higher levels of communication on nearly all sexual topics, compared with the proportions reported by young people. Secondly, young people reported higher proportions of communication with mothers than fathers. Third, the data show that parents generally talk about sexual risk prevention and developmental topics compared with experiential sex topics. Frequently discussed topics *(in bold type face)* include: (1) abstinence, HIV/AIDS, STDs, consequences of premarital sex and substance use, menstruation (girls), physical development and puberty. For instance, both parents and young people reported sexual abstinence as the most widely discussed topic. Whereas 73.6% of parents reported they had discussed abstinence with their children, child report indicated that 69.5% of mothers and 46.0% of fathers had talked to them about abstinence. Apart from topic analysis, we found that higher proportions of parents communicate more with daughters than sons on almost all topics, except for substance use.

Finally, both parents hardly discuss contraceptive and experiential sex topics with their children. In nearly half (45%) of the 20 topics, very few (less than 10%) parents reported they had ever discussed topics such as masturbation, condom use, contraception and pregnancy with their children. A comparable trend (same topics and almost the same order) was observed regarding child’s report of communication with parents (see Table 3).

Evidence from the qualitative interviews supports the above results. Topics that were frequently discussed included abstinence STDs, HIV/AIDS and premarital sex. The following statements illustrate the findings:

*“I have repeatedly told her to abstain from pre-marital sex to avoid teenage pregnancy, STDs and AIDS; because AIDS is deadly,…that it is better to abstain to avoid diseases, because AIDS and other STD’s are not written on the faces of infected persons” (Rural mother, aged 47 years).*

Reports from young people corroborated parents’ reports. The following are some quotations:

*“Among the topics my mother taught me were how to protect myself from STDs like HIV/AIDS and Gonorrhoea. She also advised me to abstain from sex to avoid unwanted pregnancy and abortion” (Urban young boy, aged 16 years).*

*“My mother talked to me about menstruation when I was about 13 years. She has also advised me to protect myself from HIV/AIDS by abstaining from sex or use condom during sex” (Urban young girl, aged 22 years).*

### Parent–child agreement

Table 3 shows the kappa coefficients for parents versus child report of mother communication on one hand, and parent versus child report of father communication with father. There were moderate to substantial agreement for parents vs. child report of mother communication. Seventeen (17) out of the 20 topics produced kappa coefficients ranging from 0.41 – 0.69. However, parent and child reports of father sexual conversation showed fair agreement, as only 5 out the 20 topics fell in the moderate agreement benchmark with reported kappa ranging from 0.41 – 0.56 (Table 3).

## Discussion

Our study focused on assessing the pattern of PCSC, sexual topics parents are generally likely to discuss with their children, and to examine triggers of sexuality communication. The study found that higher proportion of parents in the Brong Ahafo region communicates with their children about sexual and reproductive health issues. Both parent and child reports indicate that over 70% of parents had ever discussed (at least one topic) an aspect of sexual and reproductive health matters with their children. This finding is remarkable and very encouraging in the context of the Ghanaian cultural milieu, where traditionally, sexual communication is perceived to be a taboo and preserve for adults [34]. This probably may challenge the notion that sexual communication in the African context is uncommon [35].

Though most Ghanaian societies frown on open discussion of sexual issues, it does not necessarily imply that parents never talk about them at all. Discussion on them takes place as and when necessary, taking into consideration the cultural context.

Earlier studies in some African countries found a moderate amount of PCSC [4,36]. However, recent studies have found higher levels of parent–child communication. For example, Opara and colleagues [37] found that about 65% of mothers had discussed sexuality issues with their children at some point in time, while another Lagos-based study reported PCSC prevalence of 69% [38]. The present study affirms recent trends in PCSC in some parts of African.

Assessment of communication on specific sex-related topics revealed that 82.3% of parents have discussed at least a sex topic with their children, while a global single-item measure showed 74.4% of parents had done so. It appears that global single-item measure of PCSC seems to underestimate the true amount of sexual communication compared with assessment of individual sexual and reproductive health topics. The statistically significant mean difference of about 8.0 percentage difference might mean that some parents may unconsciously discuss sexual communication but rather attribute such talks as part of general communication. It is possible some parents may consider only communication episodes in which they formally sat their children down for the talks; and may discount most sexual messages that were delivered informally in censure to the child’s misbehavior.

This finding supports the evidence in the sexual communication literature that global single-item measures often result in interpretation bias [39,40], and therefore may fail to capture the all aspects of sexual communication. Similar to reports of other scholars [41,42], our study found that topic-specific communication is more effective and more preferred compared with global forms of communication.

Various triggers fuel parental communication. We found that 62.6% of parents and 59.1% of children unanimously cited “*parents’ own initiation”* as the dominant reason for parental communication action (Figure 2). The trend of responses from both participants (Figure 2) suggests agreement in reports between parents and their children. Various rationales were advanced as the motivation for pursuing sexual talks with children. Parents generally are concerned about the safety of their children. In particular, parents believe that their daughters, especially, are prone to trickeries of men, thus they need to be equipped with sex education.

We found that parental communications are directed towards a few topics. Six out of the 20 topics, that are commonly discussed by parents include abstinence, menstruation (girls), HIV prevention, STD prevention, puberty and physical development. These findings are consistent with past studies that examined discussion of sexual topics [26,43,44]. Even though this study did not explore the reasons why parents frequently discuss these topics, the general trend suggests parents’ concern about adolescent sexual safety.

Specifically, communication about abstinence from sex emerged as the common topic parents frequently discussed with their children. Nearly three-quarters (73.6%) of parents indicated they had discussed abstinence. Reporting abstinence as the commonly discussed topic is consistent with the literature [45,46]. For example, Tesso and colleagues [46] reported in their study that 84.6% young people in West Ethiopia had discussed abstinence.

Across all the 20 topics, parents reported higher proportion of communication compared to young people. As other studies have found, mothers communicate relatively more to both daughters and sons than fathers [47,48]. the different proportions reported by parents and young people imply that parent–child dyads have different estimates of how often sexual communication occurs between them, though they largely agree on topics discussed.

Unlike previous studies [39,47], our study found substantive agreement in reports of communication between parent–child dyads. At all levels of talks, mothers and children, exhibited substantial level of agreements compared to father-child discussions (Table 3). The higher level of congruence in topics discussed indicates that parents and their children were all reporting similar probabilities of occurrence of communication about the various topics. This is consistent with other studies [43], suggesting that perhaps, children are internalizing parental sexual messages. Evidence from the literature suggests that parents have a tendency to “overestimate” the degree of communication with children, while children may “underestimate” the amount of discussion parents had had with them [49]. In spite of this trend of reporting, there was no mechanism to assess from the data whether parents overestimated their report of discussion or children underestimated on their part.

The following strengths are noted in the study. First, unlike many previous African studies on PCSC that examined communication from either parent or child perspective only [1,19,37,38], our study considered reports of communication from both parents and children. It is one of the few in Ghana to systematically explore parent–child dyads’ perspectives on communication about sexual and reproductive health issues. Examining PCSC from parent–child perspectives simultaneously is important for holistic and an unbiased assessment of sexuality communication. Secondly, both sexes of parents and young people were studied, and thus provided greater insights into PCSC. This is an improvement over the many previous designs that examined sexual communication from the perspective of either parents or young people or mothers versus daughters/son. In addition, many of the previous studies covered only a limited scope of sexual communication; for example, a single topic, such as HIV/AIDS [18]. The present study comprised detailed analysis of 20 specific sexual topics, and compared with a global single item measures. The results emphasize the importance of topic-by-topic analysis of assessing PCSC.

We note some limitations in the study. Firstly, the data came from a cross-sectional study; and PCSC variables were measured retrospectively. Thus the study may suffer from recall bias since there was no mechanism to independently verify respondents’ self-reported data. However, the substantive level of correspondence between parent and child reports of communication suggests that recall bias may not be a serious problem that might refute the findings in this study. Finally, the study was conducted in the Brong Ahafo region only, out of the 10 regions of Ghana. The findings may not necessarily represent the general views of parents and young people in Ghana.

The findings from this study have implication for public health programming and research on young people sexual and reproductive health. First, the study provides information on the common topics parents are likely to discuss in sexual communication engagements with children. Thus sex educational programmes ought to target parents to expand sexual communication to cover more topics including condom and contraceptive use, which are seldom discussed. This is particularly important since condoms and contraceptive use play crucial roles in sexual risk prevention practices. Second, the study provides baseline data for future studies on PCSC. In particular, the method of assessing communication frequency by topic-by-topic analysis is could employed by in future studies since it provides a better estimate than global single-item measure of communication.

Our study explored only a few dimensions of PCSC, including the pattern, frequency and topics discussed. Future studies should consider assessment of content and quality of PCSC to examine what specific messages parent transmit to their children during sexual talks rendezvous.

## Conclusions

This study has demonstrated that majority of parents in the Brong Ahafo region of Ghana engage their children in sex-related talks, but much of the conversations is concentrated on a few sexual topics like abstinence, menstruation HIV/AIDS, STDs and puberty. Mothers talk more about sexual and reproductive health issues with theirs children than fathers. Strategies need to be devised to encourage father involvement in parent child sexual communication.

## Endnotes

^a^An adult who is not the biological parent, but whom young people describe as being like a mother or father to them, and with whom they stay with.

^b^Parent has discussed at least one sexual topic with the child.

## Abbreviations

EAs: Enumeration areas
GSS: Ghana Statistical Service
HIV/AIDS: Human immunodeficiency virus/acquired immune deficiency syndrome
IBM SPSS: International business machine statistical package for social sciences
PCSC: Parent–child sexual communication
STDs: Sexually transmitted diseases
Vs.: Versus
